# Exploring the potential use of patient and public involvement to strengthen Indonesian mental health care for people with psychosis: A qualitative exploration of the views of service users and carers

**DOI:** 10.1111/hex.13007

**Published:** 2019-11-28

**Authors:** Herni Susanti, Karen James, Bagus Utomo, Budi‐Anna Keliat, Karina Lovell, I Irmansyah, Diana Rose, Erminia Colucci, Helen Brooks

**Affiliations:** ^1^ Faculty of Nursing Universitas Indonesia Depok Indonesia; ^2^ Faculty of Health, Social Care and Education Centre for Health and Social Care Research Kingston and St Georges London UK; ^3^ KPSI Jakarta Indonesia; ^4^ Division of Nursing, Midwifery and Social Work School of Health Sciences Faculty of Biology, Medicine and Health University of Manchester Manchester Academic Health Science Centre Manchester UK; ^5^ Greater Manchester Mental Health NHS Foundation Trust Manchester UK; ^6^ National Institute of Health Research and Development Jakarta Indonesia; ^7^ Marzoeki Mahdi Hospital Bogor Indonesia; ^8^ Department of Health Services Research Kings College London London UK; ^9^ Department of Psychology Middlesex University London UK; ^10^ Department of Health Services Research Institute of Population Health Sciences University of Liverpool Liverpool UK

**Keywords:** health services, Indonesia, mental health, patient and public involvement, qualitative research, shared decision making, United Kingdom

## Abstract

**Background:**

Patient and public involvement (PPI) has the potential to strengthen mental health systems in Indonesia and improve care for people living with psychosis. Current evidence from other parts of the world demonstrates the need to understand the contexts in which PPI is to be enacted to ensure optimal implementation.

**Objective:**

To understand service users’ and carers’ views on the current use and potential applicability of PPI within Indonesian mental health services.

**Design:**

Qualitative study incorporating focus groups analysed using thematic analysis.

**Setting and participants:**

Participants included 22 service users and 21 carers recruited from two study sites in Indonesia (Jakarta and Bogor). All participants had experience of psychosis either as a service user or carer.

**Results:**

Despite the value attributed to PPI in relation to improving services and promoting recovery, current use of such activities in Indonesian mental health services was limited. Participants expressed a desire for greater levels of involvement and more holistic care but felt community organizations were best placed to deliver this because PPI was considered more congruent with the ethos of third‐sector organizations. Additional barriers to PPI included stigma and low levels of mental health literacy in both health services and communities.

**Discussion and conclusion:**

Participants felt that there was potential value in the use of PPI within Indonesian mental health services with careful consideration of individual contexts. Future aspirations of involvement enactment should ensure a central design and delivery role for third‐sector organizations. Facilitators to global collaborative research in the context of the current study are also discussed.

## 1 BACKGROUND

Patient and Public Involvement (PPI) in a mental health context is an umbrella term that encompasses a range of activities including service user movements to influence politics and change health services, knowledge generated by people living with a mental health diagnosis and the involvement of patients in the design and delivery of care.^1^, ^2^, ^3^, ^4^ Such approaches derive from a position that traditionally research and clinical decision making has been limited to the realm of intellectual and health institutions and the people that work in them to the detriment of other forms of knowledge.^5^ Advocates of this position assert that this has led to health services which at one end of the spectrum are not providing services that adequately meet individual need^6^, ^7^ and at the other are openly discriminating against the people they treat.^8^ For example, the service user movement in the UK ‘refers to the work of individuals who advocate for their personal and collective rights within the context of discrimination faced as a result of having experienced mental health difficulties and/or being diagnosed as having a mental illness’.^3^

There is a growing body of evidence for the benefits of involving service users and carers in the design and delivery of mental health services at both a systems and individual level. PPI has been shown to change services for the better^9^ through enhanced performance,^10^, ^11^ increased accountability^12^ and enhanced person‐centred care.^13^, ^14^ At an individual level, reductions in symptom severity, positive impacts on personal recovery, individual rights, mental health literacy, confidence, hope and empowerment are all associated with increased involvement in mental health services.^14^, ^15^ Such evidence has contributed to an increased emphasis on participatory approaches in mental health services internationally 16 and these principles becoming legal standards for medical care in some parts of the world.^17^, ^18^

Despite the ubiquity of involvement rhetoric within policy and practice ideologies across the world, current evidence suggests implementation remains far from optimal and service user and carer isolation and dissatisfaction persist.^10^, ^19^, ^20^, ^21^, ^22^, ^23^, ^24^ In a recent commentary, it was argued that true collaboration between people with mental health diagnoses and researchers, policy makers and health professionals cannot happen in environments which continue to perpetuate hierarchies and power imbalances albeit in a less transparent form.^25^ Often such imbalances are sustained by macro‐level factors such as the legacy of prior mental health policy and historical practice, legal frameworks and organizational cultures often not targeted or considered by PPI interventions.^20^, ^26^ Such findings have led to calls for critical examinations of such entrenched power imbalances and contexts for implementation and for PPI interventions to address these contextual factors to enable true collaboration to be realized in practice. Additional barriers include limited opportunities for involvement,^10^ diverse definitions of involvement, inadequate information provision,^21^ mental health stigma^27^ and existing practices and cultures within health services.^10^ Such implementation challenges are underexplored within low‐ and middle‐income countries (LMICs) despite the potential applicability of such approaches to improve mental health care^11^, ^28^ and the likelihood of unique challenges to meaningful implementation in these contexts.^19^ For example, the Bali Declaration (2018) written by people with psychosocial disabilities and cross disability supporters from 21 countries in the Asia‐Pacific region confirmed the relevance of inclusion to change services whilst concomitantly reaffirming the systematic and pervasive violation of people's human rights in these countries by mental health services.^29^

In Indonesia, as in other LMICs, PPI is an emerging concept, which has not been widely adopted or explored.^19^ A recent Human Rights Watch investigation into the treatment of people with psychosis revealed significant human rights violations in Indonesia, including arbitrary and prolonged hospital detention, involuntary treatment and tens of thousands of people being illegally chained up (‘pasung’) in unsanitary conditions, both in the community and in hospital settings.^30^ Such violations persist despite improvement to mental health care in Indonesia since the provision of basic community mental health care,^31^ improvements to human rights generally following the establishment of the National Commission on Human Rights in 1993 and recent changes to international covenants and domestic law which now provide an adequate legal framework for human rights protections.^32^

Mental health is now a national priority in Indonesia, and clinicians are starting to develop community‐based mental health services to support people living with psychosis. This emerging service infrastructure combined with a sustained commitment towards improving the reach and efficiency of mental health services presents a unique opportunity for PPI to shape and strengthen these emerging systems and ensure that they are designed around the needs and preferences of the people they aim to serve or to introduce alternative forms of service provision (eg third‐sector organizations).^33^ A recent systematic review drew attention to the fact that the emphasis of existing evidence is on clinical practice and professional views and identified a need for in‐depth qualitative research with patients to understanding the meaning of mental health care for those that use services in order for effective interventions to be developed and implemented.^34^ This study therefore aimed to understand service users’ and carers’ views on the current use and potential applicability of patient and public involvement activities within Indonesian mental health services.

### 1.1 Background to the collaboration

The proposal for the study was generated at a research capacity building and priority setting event in Indonesia in August 2016 funded by the British Council. A further visit to Indonesia to develop the proposal with local collaborators was funded by the ESRC Impact Acceleration Account through the University of Manchester in November 2016. Two PPI consultation events were conducted during this trip with people with psychosis and their carers to inform the study design.

## 2 METHODS

A qualitative study was undertaken utilizing focus group interviews. The choice of data collection method was informed by the study PPI advisory group. The PPI advisory group consisted of 12 people who either had lived experience of psychosis or cared for someone with a diagnosis of psychosis recruited through a partner non‐governmental organization (NGO). The advisory group was established at the initiation of the wider project^33^ and consulted on all project components. The manuscript has been prepared using the Consolidated Guidelines for the Reporting of Qualitative Data.^35^

The study formed part of a larger development award exploring the potential of involving patients, carers and communities to strengthen mental health systems in Indonesia.^33^ This four‐phase mixed‐method study aimed to develop a culturally appropriate PPI framework for use in Jakarta and Bogor, Indonesia, to strengthen local mental health systems. Phase 1 comprised of a systematic review to explore the involvement of patients, carers and communities in mental health services across South‐East Asia.^36^ Phase 2 surveyed all mental health professionals in Jakarta and Bogor to identify the important people, sources of collaboration and evidence currently used in decision making within local health services and to explore potential opportunities for involvement within the mental health system. Phase 3 explored the potential application of service user and carer involvement in mental health services from the perspectives of service users, carers, professionals and national key stakeholders using qualitative methodology. Finally, phase 4 used evidence from phases 1‐3 to inform co‐production workshops to agree priorities for a framework for use in Indonesia. The resultant framework will be used to apply for further funding to evaluate its clinical and cost‐effectiveness in Indonesia. This manuscript reports on the qualitative focus group discussions with patients and carers only. A film with more information on the study can be found here: https://www.youtube.com/watch?v=aYdX0FPvtOY%26t=2s

### 2.1 Participants and recruitment

To be eligible for inclusion in the study, participants had to either have lived experience of psychosis or have experience of caring for someone with psychosis. Additionally, they had to be aged 18 or over and have the capacity to consent to take part in a focus group discussions.

Participants were invited to take part through the voluntary and community groups they attended (see below for further information). Advertisements were displayed in community group venues for a two‐week period. Interested parties contacted a member of the research team to express interest in the study and have any questions they had about the study answered. When sufficient levels of interest were obtained, a time and date for the meeting was agreed and potential participants were notified. Participants were provided with an information sheet and consent form in Bahasa Indonesian and given the opportunity to ask again questions prior to the commencement of focus groups. All participants gave written, informed consent prior to the group starting.

A convenience sample of 43 participants consented to take part in focus groups in three community organizations (two in Jakarta and one in Bogor). Study sites were selected in relation to differing geographical, economic and urban‐rural contexts, and variety in the standard and development of mental health systems.^33^ Attempts were made to include a range of participants from both rural and urban areas. Table 1 provides more information on included study participants. More details on host community organizations can be found below:

- FG1 – Jakarta: a non‐government organization whose activities focussed on delivering information and advocacy to patients and carers about the rights of mental health consumers. Participants had significant experience of mental health activism, involvement in health services and involvement in community organizations.
- FG2 – Jakarta: participants were in receipt of services in an urban area but had much more limited experiences of mental health activism. Participants were involved in some community organizations.
- FG3 – Bogor: participants lived in a remote area where access to mental health services was much more challenging and experience of involvement in health services was minimal. Participants were involved in some community organizations.

**Table 1:** Demographic information on study participants

Focus group (FG)	Service users				Carers			
	ID	Gender (M = Male F = Female)	Age	Duration of illness (y)	Initial	Gender (M = Male F = Female)	Age	Caring role
FG 1 (NGO/Support group)	SU 1	M	32	10	C1	M	67	Father
	SU 2	M	42	10	C2	M	45	Sibling
	SU 3	M	36	15	C3	F	62	Mother
	SU 4	M	40	10	C4	F	61	Mother
	SU 5	M	30	5	C5	F	59	Sibling
	SU 6	M	28	10	C6	F	47	Sibling
	SU 7	F	46	23	C7	M	64	Father
FG 2 (Urban site)	SU 8	M	68	1	C8	M	24	Grandson
	SU 9	M	30	3	C9	F	43	Sibling
	SU 10	M	27	2.5	C10	F	26	Sibling
	SU 11	F	30	15	C11	F	32	Sibling
	SU 12	M	30	4	C12	F	53	Mother
	SU 13	F	40	13	C13	F	41	Daughter
	SU 14	M	40	1	C14	F	60	Mother
					C15	F	45	Mother
FG 3 (Rural site)	SU 15	M	41	7	C16	F	46	Mother
	SU 16	M	34	10,5	C17	F	63	Sibling
	SU 17	M	30	10	C18	F	42	Mother
	SU 18	M	22	5	C19	F	66	Mother
	SU 19	M	38	15	C20	F	70	Mother
	SU 20 IP	F	36	18	C21	M	69	Father
	SU 21	M	38	15				
	SU 22	F	38	1,5				
	Total N = 22	M = 17 F = 5	Average 38.8	Average 38 9.25	Total N = 21	M = 5 F = 17	Average 51.7	

In line with advice from the study advisory group made up of patients and carers, mixed focus groups (role/gender) were held in each location.

### 2.2 Data collection

**Figure 1:**
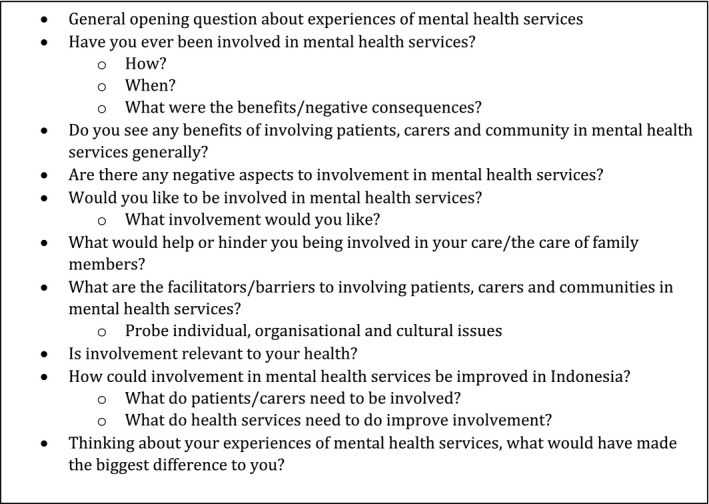
Example questions from the focus group schedule

All data were collected by Indonesian researchers supported and supervised remotely and through face‐to‐face meetings with UK and Indonesian study leads. Focus group discussions were facilitated by HS (a mental health nurse academic) in collaboration with BO (a carer‐researcher) and BK (a mental health nurse academic). An additional observer was present to support the digital audio‐recording but did not participate in the group discussions. All focus groups were held in accessible community locations in Jakarta and Bogor. Focus groups started by asking people about their understanding of involvement in mental health services generally before focusing on the current and potential use of patient and public involvement in Indonesian mental health services and the exploration of barriers and facilitators to its implementation. The focus group schedule was developed and refined amongst authors and translated into Bahasa Indonesian (See Figure 1 for example questions).

Focus groups were undertaken between May and September 2018, and lasted between 60 and 90 minutes. Groups were conducted in Bahasa Indonesian, digitally recorded and transcribed verbatim by an employee of the University of Indonesia before being translated into English for the purposes of analysis. Transcripts were anonymized at the point of transcription. In order to ensure the validity of translations, 5% of transcripts were back translated from English to Bahasa and compared to the originals in order to identify any discrepancies in meaning in line with guidelines for the undertaking of international qualitative research.^37^

### 2.3 Data analysis

Transcripts were analysed using inductive thematic analysis which involves six phases of coding and theme development.^38^ The process of analysis was underpinned by social constructionism which recognizes the complexities of individual experience and the importance of the wider context and focuses on understanding the semantic meaning attributed to people's experiences.^39^

Transcripts were first read a number of times to ensure immersion in the data. HS, HB and KJ then independently coded all three transcripts before meeting via Skype to agree a final set of codes. During this meeting, codes were organized in an iterative process which involved the removal of duplicate codes and the amalgamation of similar or related codes. Researchers also discussed any discrepancy in coding and agreed final code allocation. Codes were then organized into potential overarching themes which were considered representative of the dataset. This framework was shared with the wider study team (other co‐authors) for further refinement before agreement was reached that the identified themes fully reflected the data from all three focus groups. The final stage of the analysis was writing the manuscript which involved providing thick descriptions of identified themes and selecting quotes from the raw data to illustrate interpretations. Quotes are marked with the focus group number along with some information about the group participating in the focus group.

Preliminary analyses of the data were presented to the PPI advisory group in November 2018 to ensure any interpretations remained grounded in the lived experience of users and carers in Indonesia and their comments fed into the analysis process through the development and interpretation of data, the allocation of codes and theme development.

## 3 RESULTS

Three themes were interpreted from the data which were considered to provide a rich understanding of the potential applicability of patient and public involvement activities in Indonesian mental health services: (a) the relevance and salience of patient and public involvement in Indonesian mental health services, (b) perceived benefits and negative consequences of PPI, and (c) implementation challenges. C denotes carer participants, and SU denotes service user participants.

### 3.1 The relevance and salience of patient and public involvement in Indonesian mental health services

Of the three groups, the NGO for people with schizophrenia and their families (FG1) demonstrated the most detailed awareness of and expectations for involvement in Indonesian mental health services. The concept had less salience for the other two groups initially. This appeared to relate to the former having more direct experience of being involved in mental health services locally and nationally and of mental health activism. Participants felt that involvement was often equally as relevant at a community level (ie general public kaders – trained volunteer workers chosen by and from communities to support services at community health posts and local governments) in addition to at an individual level (ie service users and carers).

#### 3.1.1 PPI in formal health services

Despite a general desire for PPI in mental health services, there were limited current opportunities. Examples of PPI identified in health services were mostly instigated by patients or carers themselves and represented superficial levels of involvement, such as the choice of recreational activities or one way provision of information. This limited involvement reflects a potential failure on the part of mental health services to successfully implement article 19 – Living independently and being included in the community – of the United Nation's Convention on the Rights of People with Disabilities (UN) in Indonesian mental health services.

Facilitator: Were you asked what kind of therapy you want by the physicians or nurses?

C12: Nope. The medication given based on the diagnosis… like haloperidol. They never offer others. Female, 53 years, mother. (*Focus group 3/ in urban community service*)

C2: In conclusion, I think mental health care services have not engaged the community [sufficiently] yet… in the treatment plans or the support needed. It is more like we [have] got to be the one who are proactive and solve these problems with our own hands. Male, 45 years, sibling (*Focus group 1/ in support group*)

Most participants expressed a desire for greater involvement in the design and delivery of mental health care and for health services to take some responsibility for this.

Facilitator: how far… or what kind of involvement/engagement do you expect?

C11: everything, from wake up until the patient sleep at night. Families want to be involved. We need to be asked what patient's needs and wants are. Female, 32 years, sibling (*Focus group 2/ in rural community services*)

SU 5: I want to be engaged in social events like psychiatric education for the public, for example like a campaign to fight ‘pasung’ (physical restraints to a psychiatric patients in Indonesia). As a person with psychiatric disability, I want to share my experience with people in isolated areas with a lack of information about mental illness. Male, 30 years, 5 years duration of illness (*Focus group 1/ in support group*)

Some participants described how previous attempts to feedback to services about their experiences had been met with punitive responses from health professionals. For examples, complaints were interpreted as a relapse in their condition by health professionals and patients were secluded or had their medication increased as a result. Others described how involvement activity did not always bring about desired changes. Such experiences impacted on expectations and desire for future involvement in mental health services.

SU7: When I became aggressive, they will restrain me. When I complain, the doctor will add the dose. [] If we (the patients) talk too much, the doctors and nurses will take it as a relapse, so we are confused and choose to be silent. Female, 46 years, 23 years duration of illness (*Focus group 1/ in support group*)

Besides information delivery, other involvement activities identified by participants included inviting service users and carers to deliver recreational classes, including service users and carers as committee members for event organization, and engaging carers as mental health kaders for basic care and health supervision in the community.

SU4: At that hospital, I was invited twice by the Public Health Office for a gathering that was facilitated by the hospital for the patient's family. On Bipolar Day, as patients, we were also invited to share our experience and involved in a committee, for example to be a Master of Ceremony. The hospital held a Seminar, Sharing Tips and Tricks Session, once a month for patients. Male, 40 years, 10 years duration of illness (*Focus group 1/ in support group*)

Most service users and carers, however, felt that involvement in these activities was relatively superficial with limited opportunities to express ideas and decide on preferred actions.

#### 3.1.2 PPI in third‐sector organizations

Whilst few participants were satisfied with current levels of involvement in formal health services, participants were very positive about their involvement in community organizations and the support they received from these groups more generally. Reasons for this satisfaction related to there being more opportunities to talk to and share their experiences in a safe environment with people that understood what they were going through. Relationships with people working in community organizations were also considered to be improved because of reduced power imbalances and people in community groups being more approachable. Some participants directly compared the support they received from formal health services to that received from community organizations and identified a clear preference for the latter given their ability to incorporate valued activities into care and not adopting a singular focus on medication provision.

C4: Before I joined with this organization, I could not believe that my son was ill. I searched for the best psychiatrist to help my child. Finally, I found a hospital and I went there while I cried and hoped that the doctor could calm me down… by helping my child. But the doctor didn't let me know of what had happened to my child, and he said “the point is, your child is like this” the doctor said as he raised his index finger in front of his forehead as a symbol for a lunatic person. My heart was so broken, I couldn't do anything but be silent, and after he prescribed the medication I told my husband that I will never ever going back to that hospital or meet that doctor anymore. My family was not very helpful either… Finally, I met Doctor X. She informed me about this organization, so I joined. This is the right place for my child and me. The leader told me about my child's condition and I don't feel lonely anymore. When the members gather, I feel so happy like I was before [knowing my child was ill] Female, 61 years, mother

SU7: This community is not boring like hospitals. I get so many new friends that I can have conversations with, the leader also cares about me, when I get bored he will ask me to draw. Meanwhile, in hospital, all I have to do is just take the medicine, I have to wake up early in the morning… just that. I know it was for discipline but for me it was boring. Female, 46 years, 23 years duration of illness (*Focus group 1/ in support group*)

### 3.2 Perceived benefits and negative consequences of PPI

Participants acknowledged a number of benefits of PPI including sharing burden, sharing skills and experiences, improving confidence, and combating stigma. Most of these positive recounted experiences related to participants’ involvement in community organizations.

SU5: This organization is very helpful to my recovery and getting my confidence back. I’ve got so many friends here [voluntary organization]. Once, the doctor asked me to be a secretary of this organization. This makes me believe that I deserve such thing. Male, 30 years, 5 years duration of illness

SU1: An organization like this is very helpful for us. The first time my doctor diagnosed me with this illness, I felt so lonely. My friends left me. But when I joined this community group, I’ve got new friends who are dealing with the same problems as mine so we can share information. My other friends maybe will never understand about my feelings; some even have humiliated me. Male, 32 years, 10 years duration of illness (*Focus group 1/ in support group*)

Other benefits related to the role of voluntary kaders, one carer below describes how this experience not only increased her own knowledge about mental health, but also helped her neighbours to access services.

Facilitator: In these 2 months being a kader, what are the advantages that you've got?

C18: My knowledge increases, and I know what I have to do. I learned some techniques, such as deep breathing to overcome my stress, how to eliminate nervousness, relieve agitation. If I fear something, I can apply them to calm me down. Female, 42 years, mother

Facilitator: Is there any advantage to your environment or society?

C18: Thank God, I could persuade my neighbor to visit mental health service if they had problems. If my neighbor has any problem, they could talk to me. Female, 42 years, mother (*Focus group 3 in urban community service*)

Service users and carers who had been involved in PPI activities felt strongly that this experience had been important for their recovery as it gave them a sense of purpose and helped them to reintegrate into the community. Carers; however, felt strongly that PPI should not overburden family members. Whilst seeing the value of involvement in mental health services, they did not feel they should solely be responsible for care and felt that some responsibility should still rest with the government and wider community to adhere to the laws related the rights of people with mental illness. They also reported wanting support themselves from health services to support them in their care giver roles.

C2: I want the negative effects (ie in caregiving) such as feeling burdened and crisis… to be addressed by health services [] So, do not let health services have the intention to help, but we as a family become fettered. Like, like they release the chains of people with mental illness, but after that, the family becomes chained. Male, 45 years, sibling

C5: We want to provide support, but the government should also help so that the responsibility is not [just] in our hands. We want the government to be involved too. Moreover, there are already laws that regulate the rights of people with mental illnesses. Female, 59 years, sibling (*Focus group 1/ in support group*)

### 3.3 Factors affecting the future implementation of PPI in Indonesian mental health services

#### 3.3.1 Desire for PPI activities

Despite the aforementioned shared desire for PPI, participants described how other people might be unable or unwilling to engage in involvement activities for a range of reasons. These included a lack of understanding of mental health and the benefits of PPI, a lack of resources to access services and already being burdened by long‐term caregiving duties.

C5: Sometimes even the family do not know what to do, where to go, they are not at that level of knowing this. In terms of engagement, we expect that they are well educated enough so in case they see something happens about this mental illness, they won't be confused again, because the patients need to be helped as soon as possible. Female, 59 years, sibling (*Focus group 1/ in support group*)

Participants felt that service users, carers and community members may also be hesitant to engage in involvement activities because of the stigma associated with mental illness. There was a perception that in order to facilitate engagement from patients and carers, involvement activities should be offered to people which were distanced from their locality to avoid identifying them as someone with mental illness within their own community. In order to combat some of these barriers, participants emphasized the importance of offering a range of involvement activities so individual engagement could fit with personal circumstances. Community organizations were considered best placed to offer such activities.

C1: In my experience, when my son relapsed, and not all my neighbours know that my son was ill, some of them are insulting, humiliating us. Male, 67 years, father (*Focus group 1/ in support group*)

SU 16: I will join if it is in here, but I won't do anything in my neighborhood. Because I don't really know them yet. People sometimes underestimate people with mental illness. “Look, there is a crazy person” Male, 34 years, 10.5 duration of illness

Facilitator: How about the others? Would you join in neighborhood events?

SU17: No, we won't. Male, 30 years, 10 years duration of illness

C16: I am not keen to let my daughter … [to be involved in activities in our neighborhood]… I’m afraid people will think wrong about her. Female, 46 years, mother (*Focus group 3/ in urban community service*)

#### 3.3.2 Professional capacity to implement PPI

Participants felt that professionals in formal health services may not be able to enact PPI because of a perceived lack of relevance to their roles, fear it would exacerbate workload, poor communication skills and a clinical focus generally on medication. Paternalism within health services was also considered likely to further inhibit PPI.

C4: One service user told us that the nurses just gave her medicine, maybe it happened because they have limited time and energy. Female, 61 years, mother (*Focus group 1/ in support group*)

C5: We feel that health workers are often too confident and feel too much that they know everything. This means their attention to us is minimal because they already feel their service is good enough… with the services provided daily. The patients will not going to ask nurses for a chat because they are afraid of disturbing the nurses… Female, 59 years, sibling (*Focus group 1/ in support group*)

Participants felt that health professionals needed training to improve their skills in delivering information, communicating with service users and carers and providing opportunities for service users and carers to be involved in decisions about care.

C8: According to what I have seen in my brother, something that needs to be improved is communication from the professionals. Let's say he is a service user but sometimes they talk rude… sometimes they talk nice but hurtful… that's it… Male, 24 years, grandson

C9: The communication to carers should be also improved. Because the one who knows about the service users’ condition in the house is their family. Ask the family as well. Female, 43 years, sibling (*Focus group 2/ in rural community service*)

#### 3.3.3 Lack of organizational readiness to implement PPI

Participants felt that conditions in formal health services were not optimal for PPI. Reasons for this included poor co‐ordination between services and complicated bureaucracy relating to involving service users and carers within services. Nationally, participants described inadequate distribution of financial and personnel resources, the pervasiveness of stigma towards mental illness and the low political salience of mental health services at a national level when compared to physical illnesses.

C5: The bureaucracy must be simplified. Currently, if we want to do something, it is often complicated and we are asked to contact one person and then another. It takes quite a lot of time and energy so we don't want to do it. Female, 59 years, sibling

C5: In addition, mental health is not as popular as other diseases such as heart disease, HIV, etc, so that health workers may not bother. Therefore, all this time I think mental health services are still very lacking. Female, 59 years, sibling (*Focus group 1/ in support group*)

## 4 DISCUSSION

This study aimed to develop current understanding of the potential use of PPI to strengthen Indonesian mental health services for people with psychosis. A thematic analysis of three focus groups conducted with 22 service users and 21 carers identified the limited use but potential value of such activities, the significant role of community organizations in realizing PPI and driving change in Indonesia as well as a number of potential implementation challenges.

In line with research from other parts of the world, participants expressed a desire for greater levels of involvement in mental health services.^6^, ^10^, ^21^ However, in the current study carers coalesced in their concerns that any increased emphasis on involvement in Indonesia should not overburden carers or detract from Governmental or societal responsibility for the care of people with mental illness. This may be reflective of an ongoing lack of mental health service provision in Indonesia more generally^32^ despite improvements to mental health care since the mandatory provision of basic community mental health care and improvements to human rights following the establishment of the National Commission on Human Rights in 1993.^32^ Whilst involvement appears to hold potential value to Indonesian mental health services, it is unlikely to be a panacea without consideration being given to these wider contextual factors further highlighted by study participants through concerns about lack of organizational readiness to implement PPI. The health needs of carers themselves should also be addressed in addition to improving care for people with mental illnesses.^21^, ^40^

Participants in FG1 had significantly more experience of mental health activism, involvement in health services and providing care for people with psychosis. As such, the concept of PPI had greater salience to their current activities and mental health provision compared to the other two groups. Participants in all three groups however described how involvement activity was more congruent with the ethos and function of third‐sector organizations because of the increased time people had to spend with them and their perceived approachability. Some participants compared care from formal health services and community organizations directly whilst stating a preference for the latter because activities in these extended beyond medication prescription to include valued activities (such as art and creative pursuits) in line with patients in the Global North.^7^, ^41^ Any attempts to implement PPI in Indonesian mental health services should do so in close partnership with such organizations to draw on their expertise of working collaboratively with patients and carers and providing care which more adequately addresses individual needs. Service user organizations have been key drivers in the success of PPI and instigating change in other countries including LMICs and as such should be considered fundamental to PPI implementation in Indonesia.^3^, ^42^

Another finding of interest was a conceptualization of PPI which extended beyond participation at an individual level reflecting a more collectivist culture.^36^ Participants felt that the care of people with mental illness was not solely the responsibility of service users, professionals and carers, but also implicated the wider community including public figures, local government, police and neighbours. The mental health kader scheme, in particular, whereby members of local communities are trained to provide basic mental health care was viewed positively in terms of meeting the needs of service users and carers, as well as improving community relations which supports wider literature on the use of lay workforces in the South‐East Asian region.^43^ Such findings contradict the implementation of PPI in Western contexts which focus predominantly on person‐centred models. Tensions between the ethos of person‐centred care and the collective efficacy characterizing mental health activism has been identified previously in the UK as a potential reason for the failure of involvement initiatives 26 and will require careful consideration in Indonesian contexts.

Participants reported similar barriers to PPI as those in other parts of the world. These included lack of knowledge about mental health, fluctuating health status, stigma, paternalism, resource limitations, professional resistance, a need for parity of esteem between mental and physical health conditions, and lack of understanding about the benefits of involvement.^10^, ^19^, ^44^, ^45^ This identifies a potential need for national resources and guidance related to PPI in Indonesia in line with that developed by INVOLVE in the UK to address some of these identified challenges.^46^ There were a number of barriers that were of increased salience to participants in the current study including low levels of mental health awareness and high levels of stigma in health services and in communities. These barriers which have been identified previously in Indonesia have been shown to significantly impede access to care and recovery for people with mental illness.^30^ Given the importance attributed to community organizations and NGOs by participants in the current study, attention should be focused on developing creative ways to support and engage third‐sector organizations to promote mental health literacy, promote social inclusion and reduce stigma in Indonesia (eg support the development of national NGO networks and consider making NGOs an arm of psychiatry) in a way that does not diminish their unique position and strengths. Recent evidence suggests that one way to increase awareness of mental health and reduce stigma is through public engagement events which include education and art‐based activities and promote interpersonal contact between people with mental health problems and the public.^47^, ^48^ A recent evaluation of a mental health festival in Indonesia further demonstrates the potential utility of such approaches within Indonesian contexts.^49^

### 4.1 Strengths and limitations

Through its combination of focus groups and in‐depth qualitative analysis, this study has developed current understanding of the potential use of PPI within Indonesian mental health services. The results have the potential to inform the development of a culturally appropriate and need‐based PPI programme for future use in Indonesia. The study focussed solely on the views of service users and carers, and future research should explore the views of health professionals, policy makers and government officials to further enhance the use of PPI in Indonesia. Future research should also consider the use of individual interviews with service users and carers to explore some of the issues identified in the current manuscript in more depth.

The focus group discussions and qualitative analysis were enhanced through the inclusion of a carer‐researcher who co‐facilitated focus groups and contributed to the analysis of transcripts. Emergent codes and thematic frameworks were presented to an advisory group of patients and carers whose comments informed the development of final themes and ensured the analysis was grounded in the lived experience of mental health services in Indonesia. All data were collected and analysed by Indonesian researchers with UK collaborators providing qualitative supervision and guidance to support the analytical process.

All participants were from the Java region of Indonesia and self‐selected themselves for inclusion in the study having all received some form of input from mental health services. Data may therefore not reflect the views of other mental health stakeholders such as those who have not received any form of mental health service or those living in other geographical areas.

### 4.2 Reflections on global partnership working

This study represents an ongoing global collaboration between Indonesian and UK mental health academics and community organisations. This study built on previous collaborations in the form of capacity building activities and development work, and this study enabled these existing relationships to be strengthened. These relationships were of paramount importance to the success of the study and enabled the project to overcome a number of challenges including contractual delays and lengthy financial processes which significantly impeded progress. Additionally, natural disasters in Indonesia necessarily delayed data collection requiring flexibility in both approach and management. Additional facilitators to collaborative working were the inclusion of senior academics and health professionals as co‐applicants and lead researchers who could drive progress in Indonesia, the delivery of a research methods training course at study outset which was delivered to both Indonesian researchers and PPI contributors and regular supervision by Skype and during study visits.

## 5 CONCLUSION

Participants felt that there was potential value in the future use of PPI within Indonesian mental health services with careful consideration of individual contexts. Future aspirations of involvement enactment should ensure a central design and delivery role for third‐sector organizations.

## CONFLICT OF INTEREST

The authors confirm they have no conflicts of interest.

## ETHICAL APPROVAL

Approval was obtained from the University of Liverpool Research ethics committee (Ref: 2715) and the Faculty of Nursing Research Ethics Committee, University of Indonesia (Ref: No. 115/UN2.F12.D/HKP.02.03/2018).

## Data Availability

The data that support the findings of this study are available on request from the corresponding author. The data are not publicly available due to privacy or ethical restrictions.
